# A new indicator for estimating the degree of mining-induced land subsidence: the overburden’s average GSI value

**DOI:** 10.1038/s41598-023-51146-5

**Published:** 2024-01-03

**Authors:** Yaqiang Gong, Jianfeng Zha, Qingbiao Guo, Guangli Guo

**Affiliations:** 1 College of Civil Engineering and Architecture, Xinjiang University, Urumqi, China; 2 School of Environment Science and Spatial Informatics, China University of Mining and Technology, Xuzhou, China; 3 School of Spatial Informatics and Geomatics Engineering, Anhui University of Science and Technology, Huainan, China; 4 State Key Laboratory of Intelligent Construction and Healthy Operation and Maintenance of Deep Underground Engineering, China University of Mining and Technology, Xu-Zhou, China

**Keywords:** Environmental impact, Coal

## Abstract

Underground coal mining leads to land subsidence, which, in turn, results in damage to buildings and infrastructure, disturbs the original ecological environment, and hinders the sustainable development of coal mining cities. A reasonable estimation of land subsidence, on the other hand, is the foundation for building protection, land reclamation, and ecological environment reconstruction. However, when we applied the existing land subsidence estimation theory to the deep mining areas of the Ordos coalfield in western China, there was a significant deviation between the estimations and the measurements. To explain such unusual case, we propose using the overburden’s average GSI (Geological Strength Index) value instead of the compressive strength (UCS) of rock specimens for a better representation of the overburden’s overall properties. By using on-site subsidence monitoring results and historical data, we provided evidence which supports that the overburden’s average GSI value has a much greater impact on subsidence rates than the UCS. Subsequently, we investigated the relationship between three typical overburden’s GSI values and the subsidence rates via a calibrated numerical model, revealing the variation patterns of maximum surface subsidence when the overburden’s average GSI value is set at 30, 50, and 75, respectively. Finally, on the basis of the measured and simulated results, we discussed a non-conventional strip mining method for mining subsidence control in the deep mining areas of the Ordos coalfield in western China, and explained why it is possible and what are the significant advantages behind. The proposed methods, findings, and suggestions in this paper are therefore quite helpful for researchers and engineers who wish to estimate and control the mining-induced land subsidence, as well as for those who are particularly interested in the study of environment science related to land subsidence.

## Introduction

Underground longwall coal mining alters the initial stress regime of overlying rock strata (overburden), leads to the collapse of highly jointed rock mass or the bending of intact layers, and eventually causes land subsidence (i.e., mining subsidence) around the world^1–14^, which poses a serious threat to surface buildings, infrastructure, farmland, and the ecological environment if the laws of surface subsidence are not thoroughly grasped. Pioneers have clearly pointed out that the degree of mining subsidence is closely related to influential factors such as rock stiffness, mining depth, the thickness ratio of loose layers to the bedrock, etc. While such knowledge appears to be sufficient for mining practices of eastern China, we have faced significant challenges when applying it to the mining areas of western China, and all clues point to a seldom-mentioned but important influencing factor — Rock Mass Classification^15^. As China’s major coal producing areas are moving westward, the study on the relationship between mining subsidence and rock mass classification, as will be presented in this paper, is therefore necessary and significant.

The research on mining-induced land subsidence has a rather along history. In China, the classical textbook published in 1991, *Coal Mining Subsidence*^16^, had summarized the influencing factors of mining subsidence in general, including mechanical properties of the overlying strata, thickness of loose layers, dip angle of coal seams, ratio of mining thickness to mining depth, panel size, repeated mining, mining method, and roof control method. Regarding these factors, researchers have conducted more in-depth explorations in recent years. For example, Liu et al.^17^ found the high temperature generated by the combustion of coal and rock will change the properties of the overburden within a certain range, thereby intensifying surface subsidence. Zhang et al.^18^ proposed the so-called ‘local filling-caving multi-faces’ mining technique to control the strata movement and surface subsidence in the mining areas of the deep Ordos coalfield, and the major influential factors were found to be panel width, filling ratio, width of section pillar, and width of filling panel from most to least important. Xue et al.^19^ discovered that over an extended period, significant land subsidence is caused by the extraction of groundwater, and that the pace of land subsidence diminishes as time passes. Wang et al.^20^ studied solid filling mining under buildings in Tangshan, and pointed out that the compression rate is the key to surface subsidence control, and the compression rate should be kept above 80% in their case. There are many other studies^21–36^ can be found as well, yet the impact of rock mass classification has not been given due attention and discussion.

Rock mass classification , on the other hand, is a process of grouping rocks based on their physical and mechanical properties, as well as their geological characteristics. The classification system is typically used in engineering and construction projects to help determine the stability and suitability of rock masses for various purposes, such as excavation, tunneling, and foundation design. There are several rock mass classification systems in use, including the RMR (Rock Mass Rating) system^37–39^, the Q-system^40–42^, the GSI (Geological Strength Index) system^43–45^, and others. These systems typically consider factors such as rock type, strength, jointing, weathering, and groundwater conditions to assign a rating or classification to the rock mass. The classification can then be used to inform engineering decisions and design parameters. It is clear that rock mass classification takes into account not only the stiffness or strength of individual rocks, but also the overall behavior of the rock mass as a whole, which makes it potentially a more rational indicator for evaluating overburden properties and in turn for the degree of mining subsidence.

Indeed, the mechanical properties of overburden had been mentioned as the key factor affecting mining subsidence. However in the literatures^16,46^, the uniaxial compressive strength (UCS) of rock specimens is overwhelmingly used, which, in fact, only reflects a single one of the many rock parameters especially when compared to the rock mass classification systems (RMCS). This is likely the reason for the deviation in understanding the unusual mining subsidence phenomenon in the deep Ordos coalfield using traditional concepts (see Sect. "Estimating mining subsidence via rock mass classification" for details). Building on this line of thought, we initially propose an RMCS-based evaluation index (the overburden’s average GSI value), illustrate its rationality through case studies, and explain the reasons behind the unusual mining subsidence phenomenon (Sect. "Estimating mining subsidence via rock mass classification"). Subsequently, we further build a calibrated numerical model (Sect. "Methods") to investigate the relationship between overburden’s typical GSI values and subsidence rates (Sect. "Relationship between subsidence rate and overburden’s typical value"). We also discuss the applicable conditions of both the traditional evaluation indicator and the newly proposed one (Sect. "Discussion on rationality and irrationality of dividing overburden types based on rock stiffness"), and point out the underlying mechanism of the so-called 'large-scale regional strata control' from the perspective of overburden’s average GSI value (Sect. "Discussion on the mechanism ‘regional strata control’ from the perspective of overburden’s value"). By doing so, it is of great significance to bridge mining subsidence laws to rock mass classification systems to make up some incomplete understandings in the past, which will be quite helpful for researchers and engineers who wish to estimate and control mining-induced land subsidence on the basis of GSI system, as well as for those who are particularly interested in the study of environment science related to land subsidence.

## Estimating mining subsidence via rock mass classification

**Figure 1:**
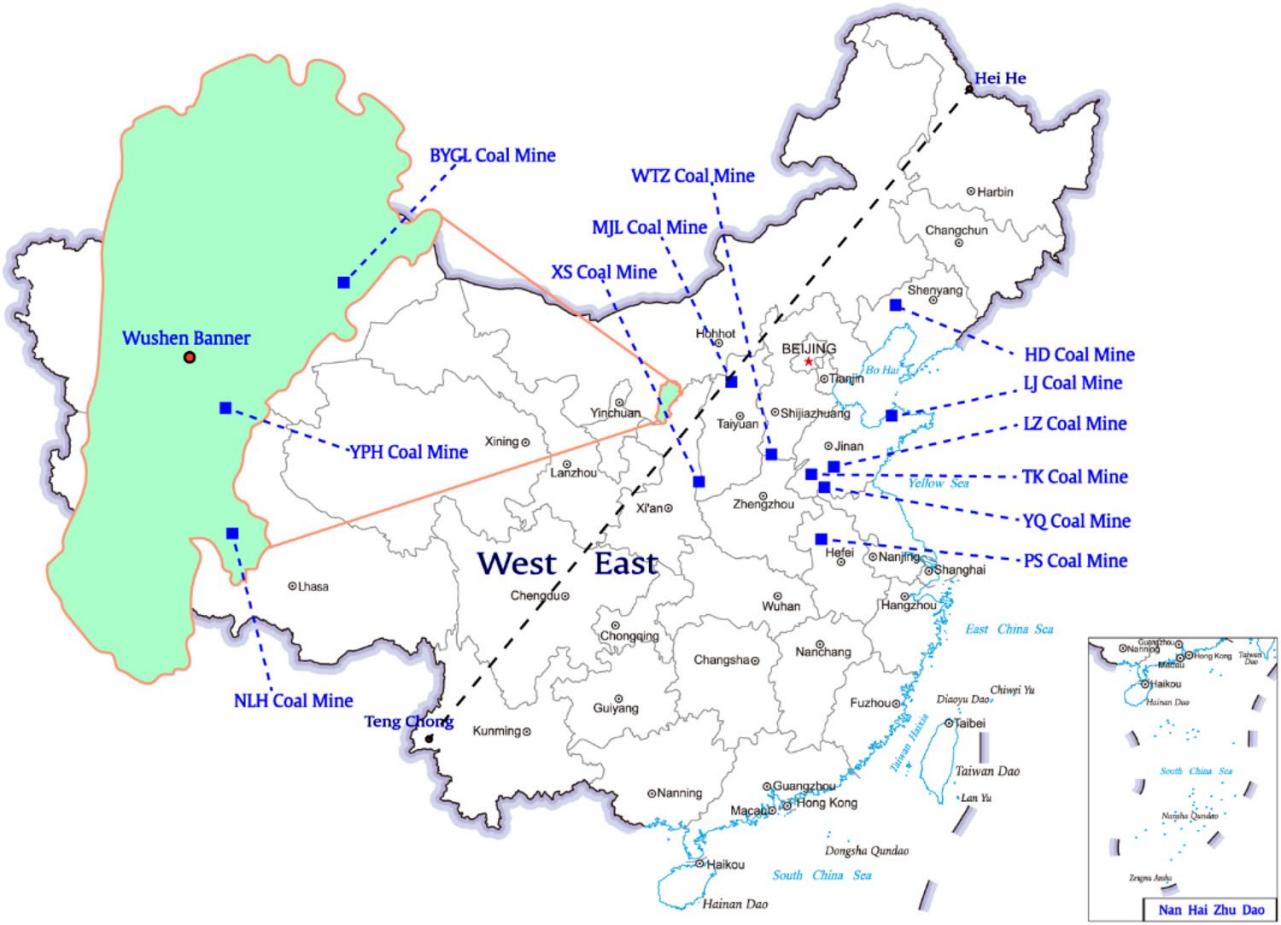
Illustration of the relative position of the coal mines mentioned in Table 1.

The study of this paper stems from three coal mines, Yingpanhao (YPH), Nalinhe (NLH), and Bayangaole (BYGL), located in Wushen banner, Ordos, Inner Mogolia Province, China (see Fig. 1). The three coal mines belong to the deep part of the Ordos coalfield, and are developed in recent five years. For those new coal mines, it is often necessary to estimate the possible subsidence values by similarity analysis for engineering design before mastering the monitoring data. Therefore, we list the basic geomining conditions of the three coal mines in Table 1 together with additional 9 coal mines chosen from historical data in the classical book^46^ to carry out the similarity analysis.

**Table 1:** Basic geomining conditions for the panels of the YPH, NLH, and BYGL coal mines. The data of the other 9 coal mines comes from the book^46^*.* The locations of the coal mines can be found in Fig. 1.

Coal mine name	Abbr	Panel name	Mining method	Average mining depth (m)	Dip	Mining thickness (m)	Panel size (length × width)	Average UCS (MPa)	Measured subsidence rate
Hengda	HD	5333	Longwall	825	6	3.3 m	2500 m × 200 m	30	0.66
Pangsan	PS	1552–3	Longwall	636	10	3.0 m	920 m × 160 m	60.4	0.77
Yaoqiao	YQ	7005	Longwall	756	11	4.8 m	1649 m × 159 m	40.2	0.21
Tangkou	TK	1301	Longwall	1012	6	3.2 m	1303 m × 208 m	–	0.82
Liangzhuang	LZ	5210–5211	Longwall	568	15	2.5 m	1000 m × 270 m	40 ~ 50	0.63
Wutongzhuang	WTZ	182102	Longwall	588	–	3.0 m	777 m × 155 m	40 ~ 60	0.40
		182101	Longwall	610	–	2.2 m	1021 m × 176 m	40 ~ 60	0.61
Majialiang	MJL	14101	Longwall	590	3	3.6 m	2849 m × 159 m	25	0.86
Xiangshan	XS	21306	Longwall	589	6	1.8 m	1880 m × 220 m	37.8	0.80
Liangjia	LJ	2408	Longwall	572	8.5	2.6 m	1260 m × 134 m	–	0.37
Yingpanhao	YPH	2201	Longwall	730	< 3°	6.5 m	2500 m × 300 m	30	Make a guess
Nalinhe	NLH	31101	Longwall	626	< 3°	5.5 m	3030 m × 240 m	35	Make a guess
Bayangaole	BYGL	311101	Longwall	620	< 3°	5.3 m	2500 m × 260 m	30	Make a guess

We can see that the panels 2201, 31101, and 311101 actually have quite similar geomining conditions. Specifically, they all adopted the longwall mining method with mining depth larger than 600 m, mining thickness larger than 5 m, panel size larger than 2500 m × 240 m, and most importantly the average uniaxial compression strength (UCS) of 30 MPa to 35 MPa. Based on this, if we first look at the panel 5333 of HD coal mine, which is closest to these three, it can be deduced that the subsidence rate of the panels 2201, 31101, and 311101, should be at least 0.7, because while their average UCS is almost the same, the mining depth of the three panels is shallower and the panel size is larger. If we then look at the panel 1552–3 of PS coal mine, it is reasonable to assume that the subsidence rate of the panels 2201, 31,101, and 311,101, should be at least 0.8, because while their mining depth is parallel, the UCS of the three panels is much smaller and the panel size is much larger. As such comparisons continue, one may finally find that it is almost not possible to obtain their real subsidence rates (i.e., 0.035, 0.093, and 0.033) based on current understanding, nor even come close to them.

The up to 24-fold difference between the measured (0.035, 0.093, and 0.033) and estimated (0.700 or 0.800) subsidence rates suggests that there must be an influencing factor that we have not clearly identified, and to the author’s opinion the rock mass classification probably is it. This is because, as we have mentioned in the Introduction, the behavior of a rock mass is not solely determined by the properties of individual rocks, but by the way in which they are oriented, jointed, and interlocked with one another. For example, a rock mass composed of stronger individual rocks (high UCS rocks) may be unstable if the rocks are poorly-jointed or fractured, while a rock mass composed of relatively weak individual rocks (low UCS rocks) may still be stable if the rocks are well-jointed and interlocked.

**Figure 2:**
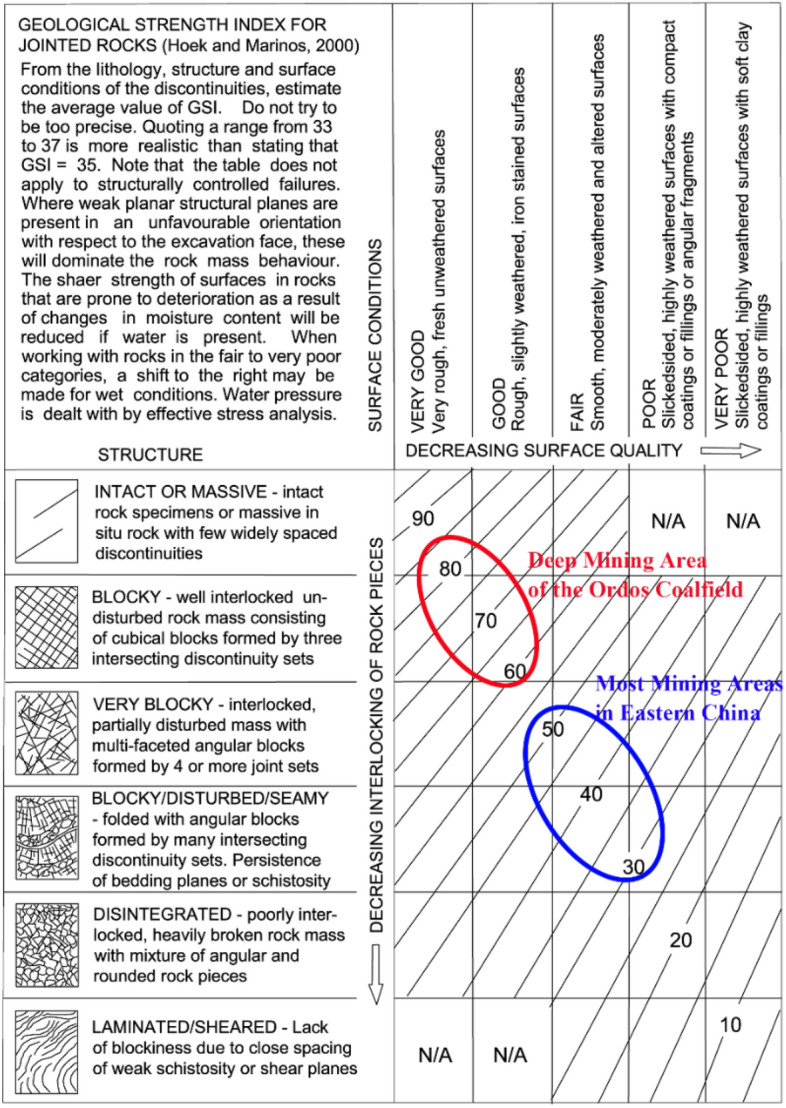
The basic GSI chart (modified after Hoek and Brown^44^) used for estimation of GSI value.

Therefore, if we see the discrepancy from the perspective of GSI (Geological Strength Index^44,47^, one of the many rock mass classification systems) for example, it will be much easier to explain. Our theory is that, in the context of similar mining depth and mining scale, the core factor that determines the degree of land subsidence is the weighted average GSI value ($\overline{{{\text{GSI}}}}$) of the overburden, and the larger the $\overline{{{\text{GSI}}}}$ value, the smaller the subsidence rate. In particular, $\overline{{{\text{GSI}}}}$ is calculated by

1 $$\overline{{{\text{GSI}}}} = \frac{{\mathop \sum \nolimits_{i = 1}^{i = n} GSI_{i} \times m_{i} }}{{\mathop \sum \nolimits_{i = 1}^{i = n} m_{i} }},$$

where ${\text{n}}$ is the total number of overlying rock strata, ${\text{m}}_{{\text{i}}}$ is the thickness of the ${\text{i}}$-th strata, and ${\text{GSI}}_{{\text{i}}}$ is the GSI value of the ${\text{i}}$-th strata, which can be estimated via the basic GSI chart (see Fig. 2). The application of Eq. 1 is data dependency, i.e., the $\overline{{{\text{GSI}}}}$ value can only be calculated when each stratum has a detailed geological description. However, it is well-known that in many cases, borehole logs are incomplete, leading to a rough estimate of the $\overline{{{\text{GSI}}}}$ value. But also note that, when there is a significant difference in the $\overline{{{\text{GSI}}}}$ values between two compared objects, a rough estimate can still reflect their divergence, at least avoiding the situation of estimating a 24-fold difference. Therefore, a rough estimate remains meaningful, and Eq. 1 can, at a minimum, be regarded as a theoretical approach for estimating the degree of mining-induced land subsidence in a more comprehensive manner.

The proposed theory can be supported by borehole logs collected from coal mines in the eastern and western China, which are available on an online database^48^. Specifically, the eastern coal mines in the database are Tangkou (TK), Daizhuang, Dongtan, Xinhe, and Xuchang, located in the city of Jining, Shandong province; the western coal mines are YPH, NLH, and BYGL. It can be found through the eastern borehole logs that terms such as ‘weathered’, ‘well-developed or extensively developed fractures’, ‘persistent bedding planes’, ‘fragmented core’, or ‘too fragmented to core’ are frequently seen, and the strata thicknesses are generally no more than 10 m, with the majority having a thickness of less than 5 m. From the 'STRUCTURE' (see Fig. 2) perspective, these characteristics should not exceed the 'Very Block' category in the basic GSI chart (see Fig. 2). When considering 'SURFACE CONDITIONS', they are likely to align with, or not perform better than, the 'Fair' category. This is especially true when taking into account that some internal strata also exhibit signs of weathering. Collectively, this suggests that the overburden’s $\overline{{{\text{GSI}}}}$ values for the eastern coal mines are likely not more than 50. Meanwhile, since the geological descriptions also document a small portion of relatively intact rock strata, the overburden is also unlikely to be classified as ‘DISINTEGRATED’. Therefore, an overburden’s $\overline{{{\text{GSI}}}}$ value of 30 to 50 should be a reasonable guess for the eastern coal mines.

On the other hand, it can also be found through the western borehole logs that the majority of strata are characterized as ‘Massive’ with only a small portion described as having ‘horizontal bedding’ or ‘wavy bedding’. In addition, strata with a thickness exceeding 10 m are quite prevalent, and terms used in the eastern borehole logs (i.e., ‘weathered’, ‘well-developed or extensively developed fractures’, ‘persistent bedding planes’, ‘fragmented core’, or ‘too fragmented to core’) are seldom seen. Based on this and referring to the basic GSI chart (see Fig. 2), it seems that the overburden’s $\overline{{{\text{GSI}}}}$ value in the western coal mines should not be lower than 60, and due to the presence of some locally developed joints and fractures within the overburden, it is also unlikely to be higher than 90.

In summary, despite our relatively rough assessment of $\overline{{{\text{GSI}}}}$ values aforementioned, it is evident that the overburden’s $\overline{{{\text{GSI}}}}$ value in the western coal mines are generally higher than those in the east, and the difference is significant. If we recall our proposed theory that the larger the $\overline{{{\text{GSI}}}}$ value, the smaller the subsidence rate, it becomes quite clear why the subsidence rates of YPH, NLH, and BYGL are significantly lower than those of the other coal mines listed in Table 1, despite their overburden’s average UCS values are very close (i.e., because the overburden’s $\overline{{{\text{GSI}}}}$ values of the coal mines in the deep mining area of the Ordos coalfield are a lot more higher). If we were to perform a similarity analysis via the proposed indicator in Table 1, we would probably not yield highly unrealistic subsidence rates such as 0.7 or 0.8 for the YPH, NLH, and BYGL coal mines.

However, knowing only the relative size of overburden’s $\overline{{{\text{GSI}}}}$ value is not enough. To more conveniently estimate the degree of mining-induced land subsidence via GSI system, one may subsequently ask, what is the relationship between the overburden’s typical $\overline{{{\text{GSI}}}}$ value and the corresponding subsidence rate?

## Methods

In this section, we take the YPH coal mine as engineering background and use the FDM (finite difference method) based commercial software, FLAC3D (Fast Lagrangian Analysis of a Continua in 3 Dimensions), to answer the question posed in Section "Estimating mining subsidence via rock mass classification" due to its many successful applications^49–51^.

### Fundamentals and model calibration

**Figure 3:**
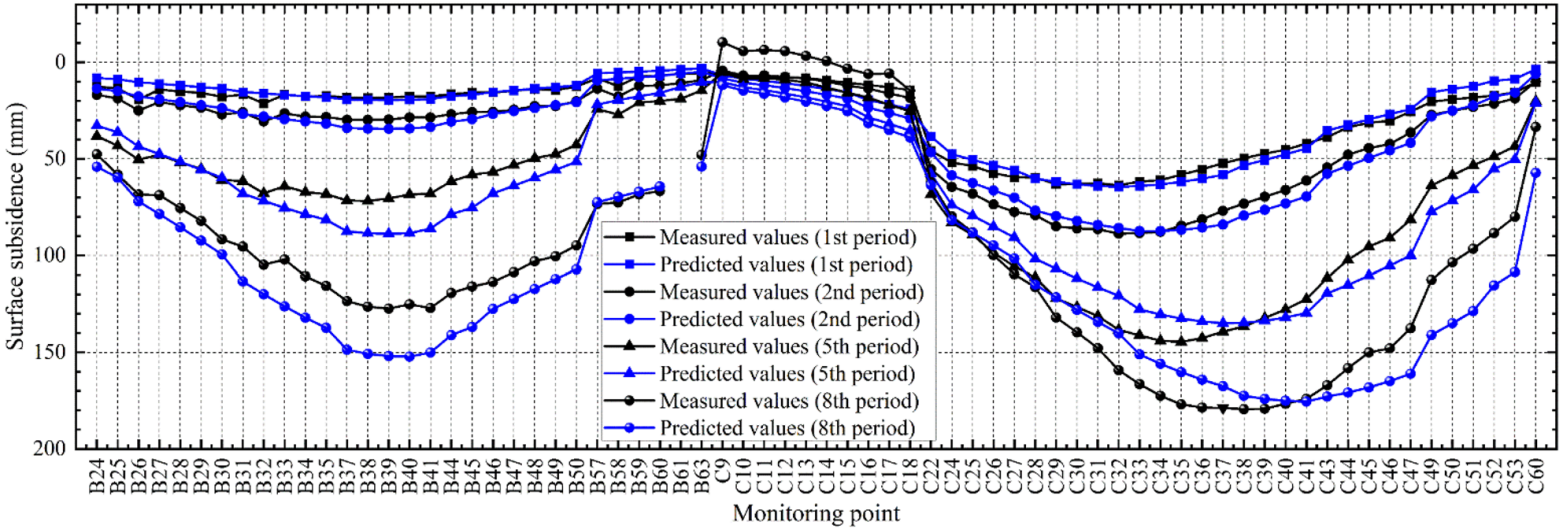
Comparison of the measured and computed subsidence values for the 1st, 2nd, 5th, and 8th periods based on the previously calibrated FLAC3D model (reproduced after Gong et al.^51^). The remaining periods (3rd, 4th, 6th, and 7th periods) are omitted because the subsidence changes are not obvious and are difficult to distinguish from the others.

In our previous study^51^, also taking the YPH coal mine as engineering background, we used the earliest surface subsidence data together with the orthogonal experiment method to calibrate a FLAC3D model. The calibrated model successfully predicted surface subsidence corresponding to several subsequent mining operations, with a maximum subsidence error of less than 10% (see the reproduced Fig. 3). Moreover, the predictions of the calibrated model also match the measured outcomes from the BYGL coal mine, which had been mined earlier than the YPH coal mine (see Table 2). Collectively, the comparison between the measurements and predictions at least proved that the previously established FLAC3D model and the corresponding rock mass parameters are within a reasonable range.

**Table 2:** Comparison between the measured subsidence rates from the BYGL coal mine and the predicted results via the calibrated FLAC3D model for the YPH coal mine.

Subsidence rate	After mining the 1st panel	After mining the 2nd panel	After mining the 3rd panel	After mining the 4th panel
Measured outcomes from the BYGL coal mine	0.030	0.290	0.370	0.500
Predicted values via the calibrated FLAC3D model	0.035	0.194	0.357	0.502

In this study, to demonstrate the impact of different overburden’s $\overline{{{\text{GSI}}}}$ value on surface subsidence, we replaced the overburden in the previously calibrated FLAC3D model with three typical ones (see Sect. "Overburden’s typical values and the corresponding rock mass properties") while keeping other conditions unchanged, to gain a macroscopic understanding.

### Overburden’s typical $\overline{{{\mathbf{GSI}}}}$ values and the corresponding rock mass properties

The determination of overburden’s typical GSI value and the corresponding rock mass properties are based on the GSI system and the Hoek–Brown equations, which have been updated for many times. In a recent edition, Hoek and Brown^44,47^ provided the basic GSI chart shown in Fig. 2, where we selected three typical $\overline{{{\text{GSI}}}}$ values (30, 50, and 75) to represent different types of overburden. Please note that, as emphasized in Fig. 2, there's no need to be excessively precise when determining GSI values, and a range of values is often more practical and realistic. Hence, $\overline{{{\text{GSI}}}}$ values of 30, 50, and 75 can actually represent rock masses with $\overline{{{\text{GSI}}}}$ values of 25 to 35 (very poor quality), 45 to 55 (average quality), and 70 to 80 (very good quality), respectively, which basically covers a variety of different geological conditions.

Another reason for choosing these three typical GSI values is that Hoek^52^ had conducted extensive research on different types of rock masses and directly provided the typical parameters for the three kinds of overburden (see Table 3). For the overburden with an average GSI value other than 30, 50, and 75, researchers who are interested in the study of mining subsidence and strata movement are suggested to use the Hoek–Brown Eqs.^44^ and the empirical parameters^47^ to obtain the detailed rock mass properties, so as to better and fully understand the laws under varying GSI values and varying geomining conditions.

**Table 3:** Three typical GSI classified rock masses (used for three different types of overburden) and their properties, provided by Hoek^52^.

Rock parameters	Very good quality overburden	Average quality overburden	Very poor quality overburden
Geological strength index	75	50	30
Hoek–brown constant	25	12	8
Friction angle	46°	33°	24°
Cohesion	13 MPa	3.5 MPa	0.55 MPa
Tensile strength	0.9 MPa	0.15 MPa	0.01 MPa
Young's modulus	42,000 MPa	9000 MPa	1400 MPa
Poisson's ratio	0.2	0.25	0.3

### Establishment of numerical models

**Figure 4:**
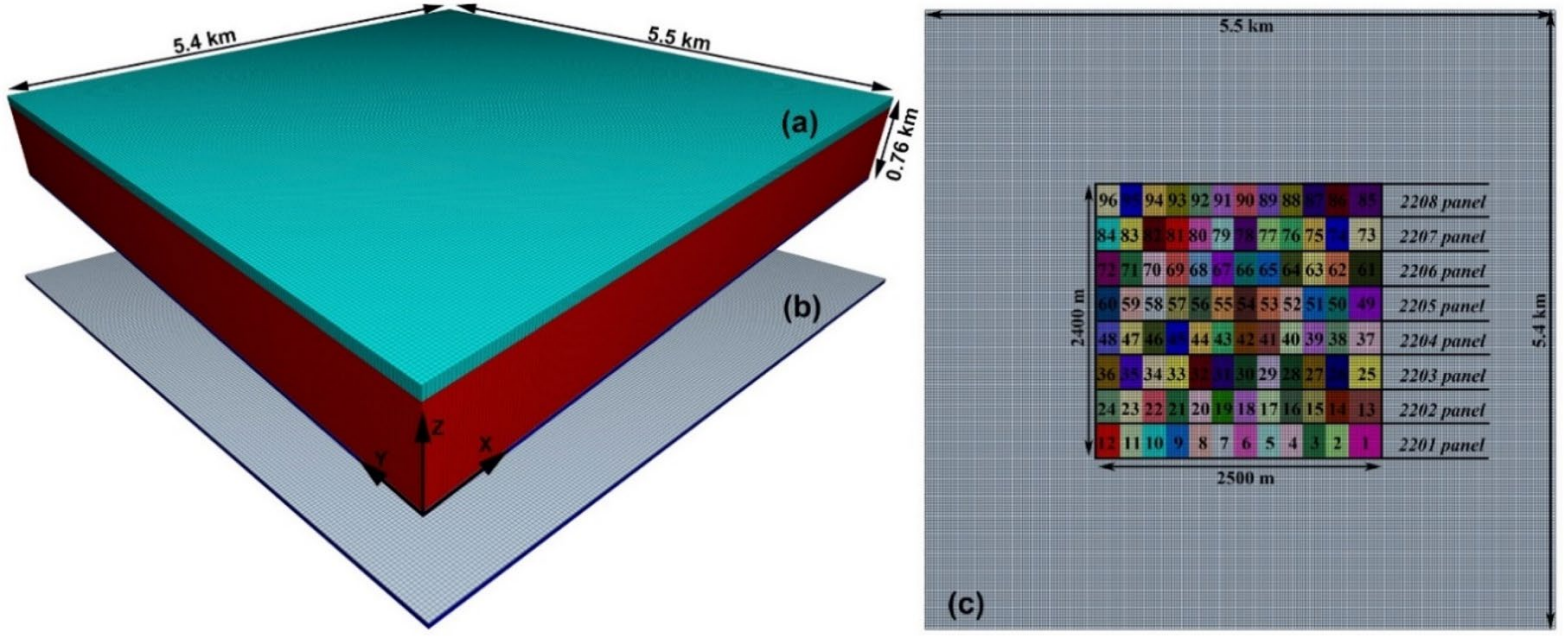
Illustration of the established FLAC3D model and schematic of the simulation scheme: (**a**) FLAC3D model; (**b**) coal seam; (**c**) mining sequence to be simulated.

We can see from Fig. 4a that the established FLAC3D model has dimensions of 5.5 km long, 5.4 km wide, and 760 m high, with the green layer for the Quaternary, the red layer for the three different types of overburden ($\overline{{{\text{GSI}}}}$ value set at 30, 50, and 75, respectively), the silver layer for the coal seam 2–2, and the blue layer for the floor rocks. All layers obey the linear elastic pre-failure behavior and the perfect plastic post-failure behavior using the Mohr-Coulomb^49^ failure criterion. The gridpoints on the four lateral surfaces are not allowed to move along the x-, y-, and z-axes, and the top is a free surface without any loads. Mesh size effect had been checked previously^51^, and it turns out that a density of 25 m × 25 m is accurate enough in this case. Statistically, the established model has a total of 3.13 million zones and 3.26 million gridpoints.

Figure 4c is a top view of the coal seam, showing the detailed mining process to be simulated, where the Arabic numerals and their corresponding colors represent the sequence and area of excavation. We can see that the simulated mining area contains a total of 8 panels, each of which is 300 m wide and 2,500 m long. Every panel is excavated 12 times, for a total of 96 excavations, and the cumulative mining length is 20,000 m. In addition, the mining area is 1,500 m away from the model boundary, which is enough to reduce the boundary effect considering the average mining depth of 730 m.

## Results and discussion

### Relationship between subsidence rate and overburden’s typical $\overline{{{\mathbf{GSI}}}}$ value

**Figure 5:**
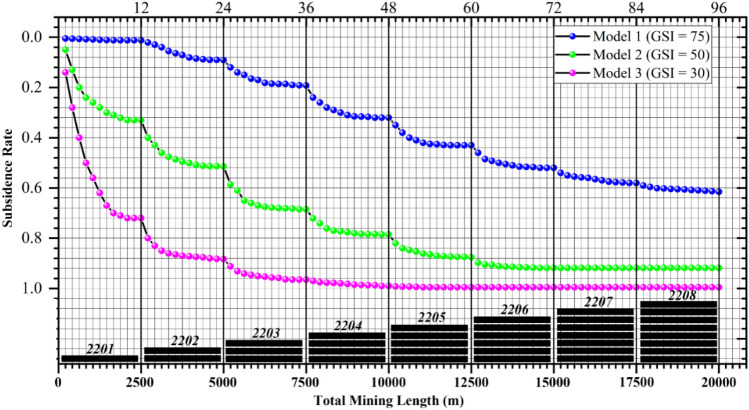
Relationship between subsidence rate and mining scale. The top coordinate axis represents the excavation times, and the numbers are consistent with those in Fig. 4.

According to the aforementioned simulation scheme, we perform excavation operations in sequence, record the maximum surface subsidence value, and present it in the form of subsidence rate in Fig. 5.

We can first see that the relationship between subsidence rate and overburden’s $\overline{{{\text{GSI}}}}$ value also relates to mining scale, and it can be very much different at varying stages. When mining the panel 2201, the maximum subsidence rate of Model 1 ($\overline{{{\text{GSI}}}}$ = 75) is only 0.012. This is quite close to that observed in the deep mining areas of the Ordos coalfield (i.e., 0.035 for YPH, 0.093 for BYGL, and 0.033 for NLH), where the rock strata are classified as ‘Massive’ mostly. Although 0.012 still exhibits a several-fold difference compared to the measured values of 0.035, 0.093, and 0.033, it is evidently more reasonable compared to the results derived based on UCS, such as 0.7 or 0.8, at least placing 0.012 within the same order of magnitude as the measured values. In addition, note that the subsidence rate (0.012) of Model 1 is lower than that observed in all the three coal mines, which suggests that the $\overline{{{\text{GSI}}}}$ value of the YPH, BYGL, and NLH coal mines may not exceed 75.

We also see that, when mining the panel 2201, the maximum subsidence rate of Model 2 ($\overline{{{\text{GSI}}}}$ = 50, average quality overburden) and Model 3 ($\overline{{{\text{GSI}}}}$ = 30, very poor quality overburden) reached 0.33 and 0.72, respectively. A subsidence rate of 0.72 is quite common in the eastern mining areas (as can be seen in Table 1), while a subsidence rate of 0.33, although less common, is also recorded in the Table. Specifically, we can find that the subsidence rate (0.33) of Model 2 is similar to that of panel 7005 (0.21) and panel 2408 (0.37). The subsidence rate (0.72) of Model 3, on the other hand, is more similar to the cases of panels 5333 (0.66), 1552–3 (0.77), 1301 (0.82), 14101 (0.86), and 21306 (0.80). Based on this, we may say that the subsidence characteristics of Models 2 and 3 collectively covered the various subsidence phenomena encountered in the mining areas of eastern China, at least for the cases listed in Table 1. Therefore, it can be inferred that the overburden type in the eastern coal mining areas may belong to the very poor quality to average quality with an $\overline{{{\text{GSI}}}}$ value possibly ranging between 30 and 50.

When mining the panel 2202 and the subsequent ones, we can observe from Fig. 5 that the subsidence rate gradually increased and eventually tend to converge during the excavation process of each panel between 1300 and 1900 m. This is more clear in Table 4, where we can see that Model 3 basically reaches the critical mining state when the width-to-depth ratio approaches between 1.23 and 1.64, not far from the range of 1.2 to 1.4 given in the textbook^16^. It seems that the empirical width-to-depth ratio of 1.2 to 1.4 is likely derived under the condition of poor to very poor quality overburden (in terms of rock mass classification), and may no longer be appropriate for the overburden of average quality to very good quality. Regarding the later scenarios, it appears, based on the properties provided by Hoek and the modeling methodology, that the critical mining state should be achieved when the width-to-depth ratio reaches between 2.05 and 2.46 for the average quality overburden, and beyond 3.28 for the very good quality overburden.

**Table 4:** The final subsidence rate after total excavation of each panel.

Panel	Width-to-depth ratio	Model 1 ($\overline{{{\mathbf{GSI}}}}$ = 75)	Model 2 ($\overline{{{\mathbf{GSI}}}}$ = 50)	Model 3 ($\overline{{{\mathbf{GSI}}}}$ = 30)
2201	0.41	0.01	0.33	0.72
2202	0.82	0.09	0.51	0.88
2203	1.23	0.19	0.68	0.96
2204	1.64	0.32	0.78	0.99
2205	2.05	0.43	0.87	0.99
2206	2.46	0.52	0.91	0.99
2207	2.87	0.58	0.91	0.99
2208	3.28	0.61	0.91	0.99

At last, we can generally conclude from Fig. 5 and Table 4 that the larger the $\overline{{{\text{GSI}}}}$ value, the slower the variation of surface subsidence rate, the larger the width-to-depth ratio as it reaches the critical mining state, and the smaller the final subsidence rate. Please also note that the above discussion is for the case of deep mining and the size of panels being 2500 m × 300 m. While this cannot cover all cases encountered in projects, it is quite beneficial for grasping some macroscopic understandings on the relationship between overburden’s typical $\overline{{{\text{GSI}}}}$ values and the corresponding subsidence rates.

### Discussion on rationality and irrationality of dividing overburden types based on rock stiffness

According to the modeling results in Section "Relationship between subsidence rate and overburden’s typical value" and rock mechanics theories, Table 5 is used to elaborate on why it is unreasonable to estimate the subsidence rate based only on rock stiffness, and where the problem lies.

**Table 5:** Relationship between overburden’s typical $\overline{{{\text{GSI}}}}$ values and rock stiffness.

Overburden’s typical $\overline{{{\mathbf{GSI}}}}$ value	Overburden type	Rock stiffness
75	Very good quality	Hard; medium-hard; soft
50	Average quality	Hard; medium-hard; soft
30	Very poor quality	Hard; medium-hard; soft; very soft

**Figure 6:**
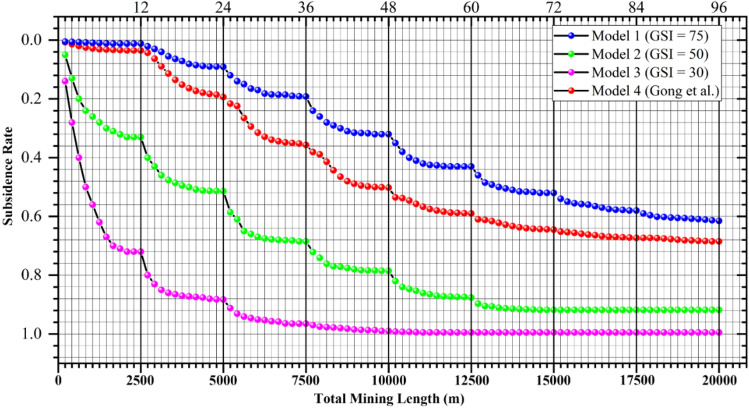
Relationship between subsidence rate and mining scale (modified after Fig. 4).

As listed in Table 5, each type of overburden can be composed of rocks with different stiffness. Specifically, the very good quality overburden can be composed of ‘hard’ rocks with UCS of 64.8 MPa. This is exactly the case provided by Hoek^52^ and investigated as Model 1 in this paper. Also, the very good quality overburden can be composed of ‘soft’ or ‘medium hard’ rocks with UCS of around 30 MPa. This is the case in the deep mining areas of the Ordos coalfield, and such rock mass is named ‘Super-Thick and Weak Cementation’ (STWC)^51^ overburden in our previous study. To demonstrate the impact of the two cases on subsidence rates, we added the subsidence variation curve under the condition of the STWC overburden (red dots) in Fig. 6. As can be seen, it is more close to Model 1 than the others, but the overall subsidence is larger, which probably due to their difference in UCS (64.8 MPa vs. 30 MPa). Therefore, based on the simulation results, it seems that the surface subsidence rate variation curve is more significantly influenced by the type of overburden, or in other words, by the overburden’s average GSI value.

On the other hand, the very poor quality overburden can be composed of ‘hard’ rocks with UCS of 60.4 MPa, such as the case of the panel 1552–3 (in PS coal mine, with panel size of 920 m × 160 m and subsidence rate of 0.77) shown in Table 1. If we recall that the subsidence rate of Model 1 (very good quality overburden composed of ‘hard’ rocks with UCS of 64.8 MPa) is only 0.01 after mining a larger size (2500 m × 300 m), we can, once again, see that it is the type of overburden or the overburden’s $\overline{{{\text{GSI}}}}$ value, rather than the UCS, that has a much greater impact on mining subsidence.

However, it is additionally important to note that estimating mining subsidence rate by comparing UCS under similar $\overline{{{\text{GSI}}}}$ value will still be meaningful. That’s probably why the method of dividing overburden into ‘hard’, ‘medium-hard’, and ‘soft’ categories had successfully guided a large amount of engineering practices in the mining areas of eastern China (i.e., because the overburden’s $\overline{{{\text{GSI}}}}$ value in the eastern coal mines are not significantly different, and under such conditions, the differences in UCS become evident, highlighting their impact on subsidence). But it is more important to stress that if there is an evident difference in the overburden’s $\overline{{{\text{GSI}}}}$ value at two locations, using traditional indicator of UCS may lead to significant errors, as discussed in Section "Estimating mining subsidence via rock mass classification".

### Discussion on the mechanism ‘regional strata control’ from the perspective of overburden’s $\overline{{{\mathbf{GSI}}}} \user2{ }$ value

**Figure 7:**
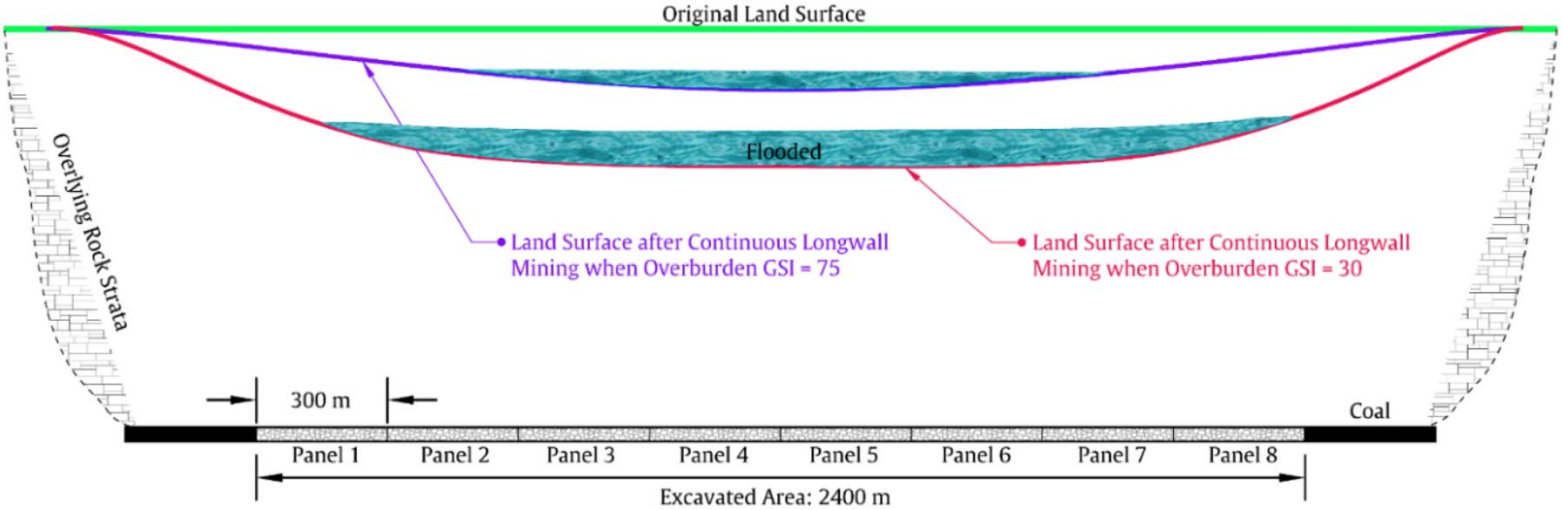
Schematic of land subsidence and flood after continuous longwall coal mining.

**Figure 8:**
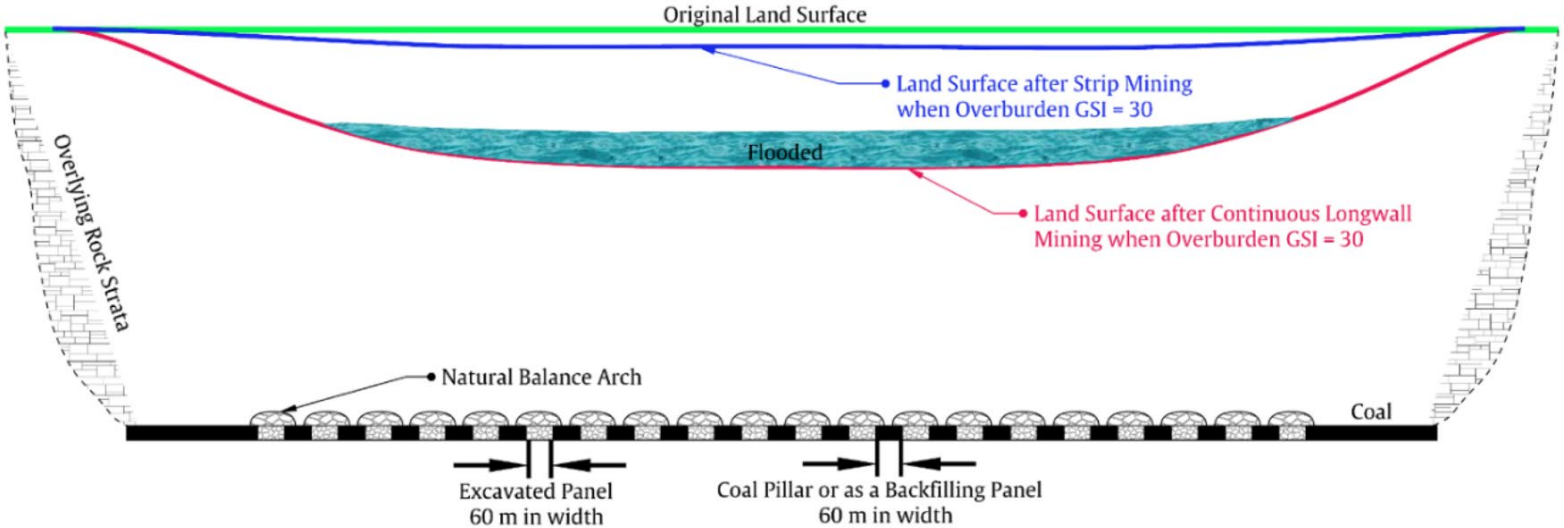
Schematic of land subsidence control mechanism behind strip mining or backfill-strip mining.

**Figure 9:**
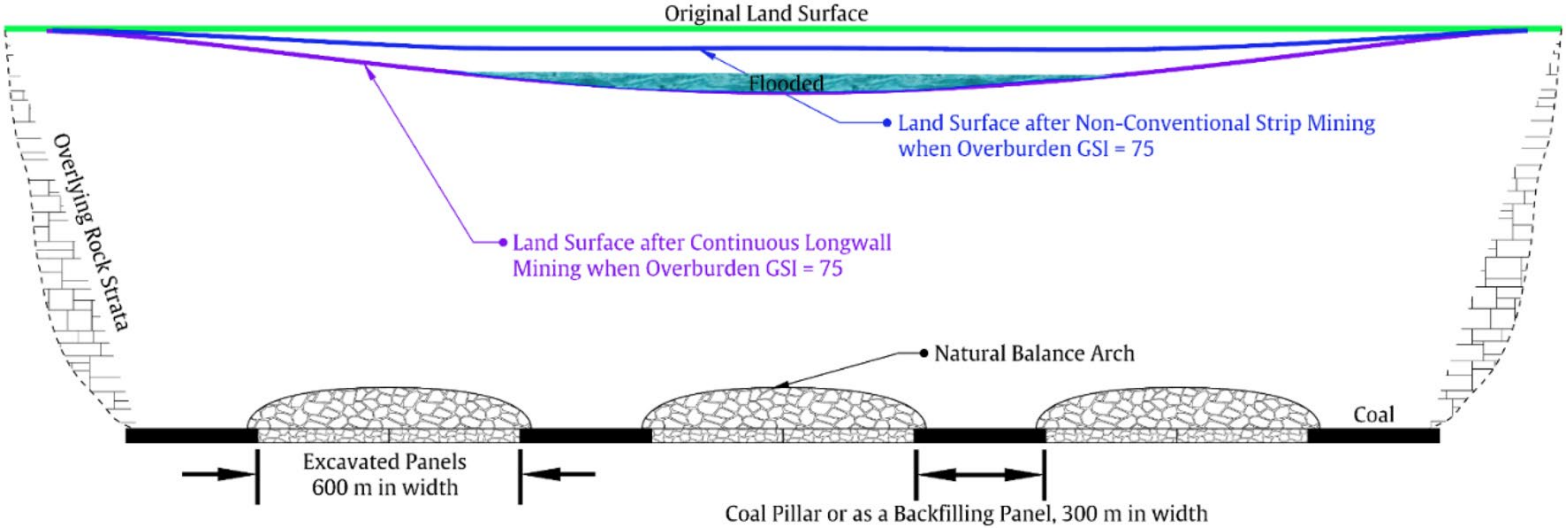
Schematic of the proposed land subsidence control method when mining under a overburden of average GSI value of 75.

The relationship between overburden’s typical $\overline{{{\text{GSI}}}}$ value, subsidence rate, and mining scale also holds a non-conventional strata control method through strip mining or backfill-strip mining, which we refer to as ‘large-scale regional strata control’. This is further discussed in this section and can be schematically illustrated in Figs. 7, 8, 9.

Figure 7 schematically shows a general picture of land subsidence and flood after continuous mining 8 longwall panels. Each panel is of 300 m in width, and the total excavated length is 2400 m. Under such scale, and according to the relationship between subsidence rate and mining scale, we have known that the subsidence rate can be 0.99 when the overburden’s $\overline{{{\text{GSI}}}}$ value is 30. Hence, if the coal thickness is 6.5 m, the original land surface will sink approximately 6.5 m to the new place indicated by the red line (see Fig. 7). A subsidence of 6.5 m is below the groundwater level in many regions of China such as the Yellow-Huaihe Plain (3.8 m in average), the Jingchu Plain (3.9 m), and the Changjiang River Delta Plain (2.5 m). Then, the subsidence basin will become a water-filled pit, greatly changing the original ecological environment. When the overburden’s $\overline{{{\text{GSI}}}}$ value is 75, on the other hand, subsidence rate can also reach 0.61, which means that the maximum land subsidence is 3.9 m, large enough to form a smaller wate-filled pit as indicated by the purple line. To reduce the damage, strip mining method, backfilling mining method, and backfill-strip mining method was proposed in the past. Backfilling mining uses gangue, fly ash, paste, and high-water material to backfill the goaf, thereby reducing the deformation space for the overlying strata and ultimately achieving the goaf of land subsidence control. However, the backfill system and materials will greatly compress the profit margin of coal mines, so it is not widely used as a conventional mining method and will not be further discussed here.

Strip mining is schematically shown in Fig. 8, where we can see that for every longwall panel being excavated, a strip coal pillar is left behind. Please note that, when the overburden’s $\overline{{{\text{GSI}}}}$ value is 30, the panel width is only 60 m, because the natural balance arch above the goaf is difficult to stabilize and subsidence will increase rapidly if the panel width becomes larger (recall that subsidence rate of Model 3 will be 0.72 when panel width is 300 m). Such cases can be found in Shandong Province of China when mining under overpass such as the panels 8301 to 8307 of Daizhuang coal mine. However, strip mining will result in the waste of nearly half of the coal resources, and it also faces the problem of more frequent relocation of mining equipment. Specifically, for the case in Fig. 7, the mining equipment needs to be relocated 7 times, while for the situation in Fig. 8, it needs to be relocated 19 times. Regarding backfill-strip mining, it is quite similar to strip mining, and the major difference is that the coal pillars left in the strip mining will also be mined using backfilling mining. The mechanism behind them is basically the same, so the land subsidence control of backfill-strip mining will not be elaborated here.

Most importantly, there will be a big change when the overburden’s $\overline{{{\text{GSI}}}}$ value is 75. This is schematically illustrated in Fig. 9, where we can see that the proposed land subsidence control method is similar in form to strip mining. But it should be emphasized that significant differences exist. Specifically, the conventional strip mining method usually mining one panel, followed by leaving one coal pillar, and the panel width and pillar width are very close, often not exceeding 100 m. However, when the overburden’s $\overline{{{\text{GSI}}}}$ value reaches 75, we can leave a coal pillar after mining two or even three panels, and each panel can have a width of 300 m. This is because that, for a ‘$\overline{{{\text{GSI}}}}$-75-Overburden’, the subsidence rate is only 0.01 after mining one panel of 300 m width, is 0.09 after mining two adjoining panels, and is 0.19 after mining three panels. In other words, because of the ‘Massive’ characteristic of the overburden having a average GSI value of 75 (e.g., the mining cases in the deep mining areas of the Ordos coalfield), the natural balance arch formed there will be much stronger and the span of the arch can be very large.

The above differences make the proposed subsidence control method have additional advantages. Firstly, because the total mining width is 2 to 3 times the width of the coal pillars left, the coal recovery rate can be increased from about 50% to 66% or even 75% depending on the overburden’s average GSI value. Secondly, because the width of each panel is relatively large (e.g., 300 m vs. 60 m), the number of mining equipment moves can be reduced from 19 to 5 times, which greatly improves production efficiency. Meanwhile, the coal pillars left can also be re-mined using backfilling mining method to further improve the coal utilization rate. Therefore, the subsidence control method proposed is not a conventional strip mining or backfill-strip mining method. Considering that the overburden structure formed in this way is connected over a large area, we call it a ‘large scale regional strata control’ method. The mechanism behind this is to fully utilize the bearing capacity of the high $\overline{{{\text{GSI}}}}$ value overburden (especially the ‘Massive’ type with $\overline{{{\text{GSI}}}}$ value higher than 75), as just discussed. But it should be noted that doing so also places a higher load on the support bodies and in turn may induce mine tremors or rock burst phenomena, which merits further studies.

## Summary and conclusions

This paper stems from the significant deviation between the estimated land subsidence rates using traditional indicator of UCS and the measured ones in the deep mining areas of the Ordos coalfield. To solve this, we proposed a new estimation indicator, the overburden’s average GSI value, to better represent the overburden’s overall properties. By analyzing 12 cases both in eastern and western China, we provided evidence to support our proposal. Subsequently, we investigated the relationship between subsidence rates and three typical overburden with $\overline{{{\text{GSI}}}}$ value set at 30, 50, and 75, respectively, thus providing reseachers with a reference framework. We also discussed the relationship and applicability conditions between the traditional indicator and the newly proposed one, and finally discussed an unconventional strata control method from the perspective of overburden’s average GSI value. The main conclusions of this paper are:

1. The overburden’s average GSI value, as a newly proposed indicator for estimating the degree of mining-induced land subsidence, successfully explains the unusual extremely low subsidence rates observed in the YPH, BYGL, and NLH coal mines, which appears to be more comprehensive than the traditional indicator of UCS.
2. According to the borehole log database and the modeling results, the overburden’s $\overline{{{\text{GSI}}}}$ value in the mining areas of eastern China probably falls within the range of 30 to 55, while that for the mining areas of the deep Ordos coalfield should be between 60 and 75. The much smaller land subsidence rate observed in the YPH, BYGL, and NLH coal mines is essentially because of their much higher overburden’s $\overline{{{\text{GSI}}}}$ value.
3. When the overburden’s $\overline{{{\text{GSI}}}}$ values in two locations are relatively close, it is reasonable to use the traditional indicator of UCS to estimate subsidence rates. However, when there is a significant difference in their overburden’s $\overline{{{\text{GSI}}}}$ values in two locations, using the UCS still may lead to considerable errors.
4. The ‘Massive or Intact’ rock mass dominated overburden in the deep mining areas of the Ordos coalfield is very well-suited for using a non-conventional strip or backfill-strip mining method (large scale regional strata control) by taking full advantage of the overburden’s high $\overline{{{\text{GSI}}}}$ value.

## Data availability

The data that support the findings of this study are available on request from the corresponding author, and are partly available on the online database (https://data.mendeley.com/datasets/vcpz47r3sv/2).
